# Public acceptance of default nudges to promote healthy and sustainable food choices

**DOI:** 10.1186/s12889-023-17127-z

**Published:** 2023-11-22

**Authors:** Dominic Lemken, Simone Wahnschafft, Carolin Eggers

**Affiliations:** 1 Institute for Food and Resource Economics, University of Bonn, Nußallee 21, 53115 Bonn, Germany; 2 Research Training Group in Sustainable Food Systems, University of Göttingen, Heinrich- Düker-Weg 12, 37073 Göttingen, Germany; 3 University of Göttingen, Göttingen, Germany

**Keywords:** Default nudge, Food choices, Public acceptance, Perceived intrusiveness, Perceived effectiveness, Transparency

## Abstract

**Background:**

Default nudges are an increasingly prominent tool for promoting healthy and sustainable food choices; however, questions of acceptance remain. While default nudges are more acceptable to the public than traditionally paternalistic tools that aim to restrict choice, they are also the least acceptable amongst nudging strategies. Little research has investigated the aspects of default nudge design that can be leveraged to better uphold freedom of choice, increase public acceptance, and therefore heighten legitimacy of default nudges. Consequently, this study examines public acceptance of five food choice default nudges with demonstrated precedent of effectiveness, as drawn from research studies and/or real-world policies, along with a design variation of each anticipated to increase acceptance. Three drivers of acceptance – perceived intrusiveness, perceived effectiveness, and own behavior – are examined.

**Methods:**

An online survey was administered in Germany (N = 451) to a sample representative of the adult population on quotas of age, gender and income. Acceptance and drivers were measured using seven-point Likert scales. Significant differences in median acceptance of the nudge were determined and displayed graphically. Ten proportional odds ordered logit models were applied and estimated using a maximum likelihood approach to investigate the mechanisms of nudge acceptance.

**Results:**

Examined changes in nudge design, particularly decreasing costliness of opting out and increasing transparency, increased the acceptance of three of the five nudges (N2.2: p = 0.000; N3.2: p = 0.000; N4.2: p = 0.008). Perceived intrusiveness emerged as the most prominent driver of acceptance (negative relationship), followed by perceived effectiveness (positive relationship). Own engagement in the target behavior of the nudge and socio-demographic variables demonstrated negligible impact on acceptance.

**Conclusions:**

Mitigating the costliness of opting out and improving nudge transparency emerge as key opportunities for choice architects to improve public acceptance, and thereby potentially identify ‘sweet spots’ in designing default nudges that are both effective and acceptable. The protection of individual freedom of choice and effectiveness are key aspects for choice architects to communicate to increase acceptance.

**Supplementary Information:**

The online version contains supplementary material available at 10.1186/s12889-023-17127-z.

## Background

In a concerted effort to integrate the health and sustainability agendas for food system transformation, the EAT Lancet Commission published the planetary health diet (PHD) in 2019, establishing the first scientific targets for a dietary pattern to promote both healthy diets and sustainable food production on a global scale by 2050 [1]. Meeting the PHD targets in most industrialized countries will require stark increases in the consumption of fruits, vegetables, nuts, wholegrain cereals, and unsaturated fatty acids, as well as decreases in the consumption of meat, dairy products, saturated fatty acids, and sugars [1].

To achieve such shifts, governments have at their disposal several behavior change interventions to promote population-level behavior change. One framework that is commonly used to taxonomize these interventions is the Nuffield Ladder of Intervention, which introduces individual freedom to choose as a key guiding concept [2]. Namely, the ladder distinguishes between ‘soft’ interventions (I.e., those on the lower rungs of the ladder), such as information and education, which infringe the least on individual choice and ‘hard’ interventions (I.e., those on the top rungs of the ladder), such as mandatory standards or bans, which intrude most heavily on individual choice. Following the foundational liberal values underpinning the ladder, the general principle for policymakers to follow is that, when possible and effective, soft measures are to be preferred over hard ones.

In the arena of policymaking for shifting food choices for health and sustainability reasons, most governments to date have favored the use of soft interventions [3]; however, these interventions have often been found to be either (a) ineffective at promoting long-term behavior change, particularly compared to interventions higher on the ladder; or (b) effective at promoting behavior change amongst those who are already better positioned in society to achieve the desired behavior change, thereby generating inequities along socioeconomic lines [4, 5]. One of the key reasons that has been posited for persistent reliance on soft interventions, despite evidence of low effectiveness, is the issue of acceptance: acceptance of hard interventions, which impinge more heavily on individual freedom of choice, may be low amongst several relevant stakeholders [4]. A systematic review of studies on public acceptance of policies to shift health-related behaviors offers support for this rationale, finding low public acceptance of interventions higher on the Nuffield Ladder relative to those interventions lower on the ladder [6, 7]. Low public acceptance is also inextricably linked to low policymaker acceptance, particularly in democratic contexts in which policymakers must navigate acting in the public interest while maintaining public favor for re-election.

It is in the context of this effectiveness-acceptance trade-off where the appeal of Thaler’s and Sunstein’s *nudge* can be easily understood. Thaler and Sunstein essentially posit that it is possible for governments and implementing institutions to effectively change behavior while maintaining individual freedom of choice. Such a balance may be achieved by use of a nudge, which refers to a shift in the way choices are presented to decision-makers (I.e., the choice architecture) that predictably alters behavior in the population without barring any options or significantly changing economic incentives [8]. In little over a decade since its first inception, nudging has already become a prominent consideration in the policymaking toolbox, as many governments and international development agencies have integrated ‘nudge units’ to guide policy and operational decision-making [9].

Growing evidence points to one particularly effective nudging strategy: the default nudge [10]. Default nudges, which have been highlighted for their potential to promote healthy and sustainable food choices across several studies [11–13], refer to a particular type of nudge in which the ‘default’ option - i.e., the outcome that arises when a decision-maker does not make an active choice – is altered by a choice architect to promote a shift in behavior.

While default nudges are a very promising tool from an effectiveness standpoint, questions of acceptance remain. Namely, while default nudges have been found to be relatively more acceptable to the public than more traditionally paternalistic tools that aim to restrict or eliminate choice [14], default nudges have also been found to be the least acceptable to the public amongst nudging strategies [15, 16].

Public acceptance has been raised as a key consideration in designing ethical nudges, as it serves as a proxy to understanding the extent to which each nudge aligns with the preferences of the population impacted by the nudge and thus the extent to which each nudge is legitimate [17, 18]. Indeed, while nudging first emerged with a promise to find the ethical ‘sweet spot’ in shifting behavior without infringing on individual freedom to choose, several objections have been raised by critics on the extent to which nudges really do so, particularly if they prey upon cognitive biases and heuristics in such a way that individuals end up choosing options that run counter to their actual preferences [17].

It is also of fundamental importance to understand the mechanisms underpinning public acceptance, or lack thereof. This importance draws from communication research, particularly the theory and empirical evidence for the effect of framing, defined as ‘the process by which a communication source constructs and defines a social or political issue for its audience’ [19]. Namely, the specific conceptualizations that are used to *frame* policies have been found to exert an, albeit moderate, influence on public attitudes towards those policies across several policy arenas, including those related to promoting healthy and sustainable food choices [20, 21]. Thus, understanding the factors associated with acceptance offers insights for levers that can be acted upon in the communication of a nudge to increase public acceptance.

Given the salience of public acceptance in designing successful nudges that carefully navigate the effectiveness-acceptance trade-off, this study aims to investigate public acceptance of a series of nudges designed to promote healthy and sustainable food choices amongst consumers in Germany. Germany makes for an applicable study context, as Germany has been highlighted as a pioneering country in the application of behavioral insights, with a ‘nudge unit’ based within the Federal Chancellery since 2015 [22]. In addition, public acceptance of health nudges in general has been found to be quite high in Germany [23], a context with limited adoption of more traditionally paternalistic nutrition policy instruments despite a persistently high burden of diet-related disease [24, 25]. This study is guided by two research questions, each expanded upon below.

**Q1. What design changes improve public acceptance of default nudges for promoting healthy and sustainable food choices?**

**Fig. 1:**
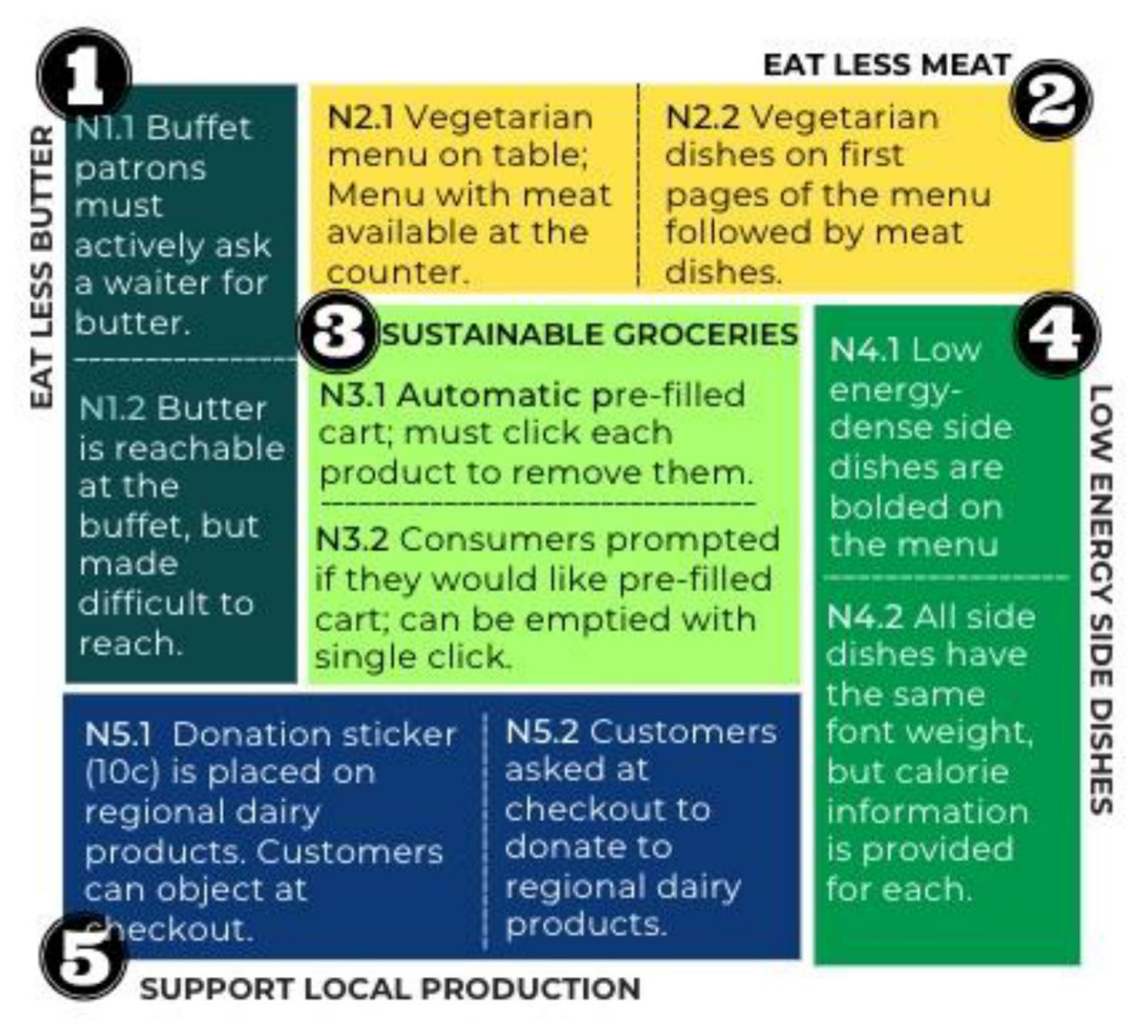
Summary of five default nudge scenarios and respective variations examined

Given the understanding that nuances in nudge design carry large implications in terms of acceptance, and thereby legitimacy, of nudge adoption [26], this study explores the effect of shifts in the design of nudges on public acceptance. Specifically, five nudge scenarios are evaluated, as well as one variation of each nudge in which an element of the nudge design is varied (see Fig. 1). The selected nudges were adapted from nudges that have been demonstrated in the literature to be promising from an effectiveness standpoint for promoting various healthy and/or sustainable food choices. All but one (nudge 4) can be classified as default nudges. For each of the nudges, the second variation is anticipated to increase acceptance.

**Q2. How do perceived effectiveness, perceived intrusiveness, and engagement in the targeted nudge behavior influence the acceptance of default nudges for promoting healthy and sustainable food choices?**

This study investigates the influence of three mechanisms on public acceptance of the five proposed nudge scenarios and their variations. These mechanisms were selected based on the following two criteria: (a) they are highlighted in the literature as particularly prominent drivers of nutrition policy acceptance amongst the public; and/or (b) if found to play a role in acceptance of default nudges, they are actionable levers for improving the communication of default nudges to increase acceptance. The first mechanism, which captures the extent to which the public believes the default nudge to be effective at achieving the desired shift in behavior, has been found to be one of the strongest predictors of nutrition policy acceptance in previous studies [27, 28], including specifically for nudges to shift food choices [29, 30]. Perceived intrusiveness, or the extent to which people believe the default nudge to limit freedom of choice, is another salient mechanism that has been found to mediate acceptance of a range of nutrition policies [26, 28, 31]. Finally, this study examines the impact of self-reported engagement in the behavior that is targeted by each nudge, as this has also been found to mediate nutrition policy acceptance [6].

## Methods

### Study procedure

Following a few socio-demographic questions for the purposes of quota sampling, participants were asked to evaluate five nudge designs, as well as a variation for each nudge design. Each nudge scenario followed an identical procedure. First, participants were asked how they typically behave in a specific setting, such as whether they typically consume butter at a restaurant buffet when the following nudge scenario focused on butter consumption. Then, participants were briefly introduced to the nudge scenario in a descriptive manner to avoid influencing perceptions. Participants were subsequently asked to rate their “acceptance” of the nudge scenario on a seven-point Likert scale (ranging from − 3 “full rejection” to 0 “indifferent” to + 3 “full acceptance), as well as their perceived freedom to choose, whether they believed the nudge would effectively change their personal behavior, and whether they believed the nudge would effectively change the behavior in general. The perceived effectiveness on personal behavior was dropped from the data analysis because the relationship with acceptance is mediated by the perceived effectiveness in general (bivariate correlations ranging between 0.3 and 0.85). The same evaluation was then conducted for the variation of the nudge scenario to compare the scenarios. The order in which the five nudge scenarios were presented to participants was randomized to avoid ordering effects. However, the variation of a nudge scenario always followed the original nudge scenario. For a full summary of statements used to measure mechanisms underpinning acceptance, see Supplementary Materials (Table S1).

### Overview of default nudge scenarios

*Nudge 1. Eat Less Butter.* The first nudge was drawn from a study conducted amongst students in Denmark, in which a shift in the positioning of butter at a buffet from easily within reach of consumers to available only upon request was found to effectively decrease uptake from 0.7 to 0.3 butter packages consumed per person [32].

*Nudge 2. Eat Less Meat.* The second nudge was adapted from a study conducted on the campus of a large university in the United States, in which the provision of a default menu with vegetarian options was found to increase the choice of vegetarian meals in a school canteen amongst recipients compared to conventional menu options (OR = 4.10) [11].

*Nudge 3. Climate-Friendly Groceries.* The third nudge investigated examines the acceptance of a pre-filled climate-friendly grocery cart in an online supermarket setting. The precedent for the effectiveness of this default nudge was demonstrated amongst low-income consumers in the U.S., in which randomization to a pre-filled nutritionally balanced online grocery cart was found to decrease total calories and energy density of purchases amongst recipients over the course of five weeks compared to a control group [12].

*Nudge 4. Low Energy Density Dishes.* The fourth nudge draws upon the results of manipulations to a restaurant menu conducted by Dalrymple et al. in a U.S. theme park, in which increasing the font weight and centrality of low energy side dishes on a menu increased selection of low-energy side dishes to 42.2% compared to 18.1% in the normal menu with all side dishes displayed the same [33].

*Nudge 5. Donation for Regional Dairy Products.* The fifth and final nudge concerns generating support for local dairy farmers by way of a default donation sticker placed on dairy products, which can be opted out of by way of an in-store coupon in a supermarket setting. This nudge was drawn from a real-world policy adopted by one grocery store chain in Sweden in 2015 that generated an extra 28,000 krona (~ 2.500 EUR) per dairy farm in donations over just 6 months [34].

A summary of the variations of each nudge examined, as well as the design element varied across the variations, can be found in Table 1.

**Table 1:** Summary of five nudge scenarios, variations, and design element varied across variations

Nudge	Variation 1	Variation 2	Design element varied
1. *Eat Less Butter*	Patrons must actively ask a waiter for butter.	Butter is reachable for patrons at the buffet, but it is made to be difficult to reach.	Shift in nudge intrusiveness by decreasing the ‘social’ cost of opting out.
2. *Eat Less Meat*	A vegetarian menu is placed on the table. A normal menu with meat options is available but must be actively fetched at the counter.	A menu with both meat and vegetarian meal options is placed at the table; however, vegetarian dishes are placed on the first page of the menu.	Shift in nudge intrusiveness by decreasing the ‘physical’ cost of opting out.
3. *Climate-Friendly Groceries*	Consumers are automatically provided a pre-filled cart and must click products individually to remove them if they are not desired.	Consumers are presented with a choice about receiving a pre-filled cart, which can be emptied with a single click.	Increased nudge transparency.
4. *Low-Energy Side Dishes*	Low energy dense side dishes are bolded on the menu.	All side dishes have the same font weight, but calorie information is provided by each side dish on the menu.	Shift from a salience nudge to an information nudge.
5. *Donations for Regional Dairy Products*	A 10-cent donation sticker is placed on regional milk products, to which customers must actively object at checkout.	The cashier asks the customer if they agree to a donation on regional milk products in their cart at checkout.	Shift in nudge intrusiveness from a default structure to a forced active choice.

### Data analysis

To answer the first research question, acceptance between the original scenario and variation are compared, as well as displayed graphically to visualize the effects of the variation on full refusal, indifference and full support. In addition, we apply a median test for equality of matched pairs of observations, previously explained by Snedecor and Cochran [35]. The null hypothesis is that the median of the differences is zero; no further assumptions are made about the distributions. The null hypothesis is rejected for p-values smaller than 0.05. The test speaks to the probability of one nudge variation being more accepted than another. I. For the second research question regarding the mechanisms of nudge acceptance, ten proportional odds ordered logit models are applied. The models are estimated using the maximum likelihood approach. Such a model can be thought of as multiple binary logistic regressions on the relative probability to be in one category rather than the next lower one [36]. The explanatory variables have been standardized to compare. Odds ratios (ORs) are presented graphically. The value of “1” implies no OR change across the values of the independent variable. The model for all 10 scenarios is presented within a single table. All models control for sociodemographic characteristics of consumers.

### Participants

451 participants completed the survey (see Table 2). They were recruited by a market research firm to be representative of German consumers on quotas of age, gender and income. The survey was pre-tested amongst 50 participants from different educational backgrounds. To minimize selection bias, participants received minimal information on the survey content prior to participation. To ensure data quality, attention checks were included in the survey and participants who failed were unable to complete the survey. In addition, participants who took less than 5 min (approximately half of median time, one third of mean time) to complete the questionnaire, were excluded, as it is assumed that they did not have time to adequately process and evaluate the scenarios. The cleaned data set includes 409 participants.

**Table 2:** Sample description and quoted variables

Variable	N (409)	Freq [%]		Pop. [%]
**Gender** Female	219	53.7		50.9
Male	189	46.3		49.1
**Age** 18–24	10	2.4		11.1
25–34	83	20.3		19.1
35–44	95	23.2		18.0
45–54	79	19.3		21.8
55–64	97	23.7		20.9
65–70	45	11		9.1
**Income (Euro, Monthly Net)** <900	20	4.9		4.9
900–1300	32	7.8		8.4
1301–1500	19	4.6		4.5
1501–2000	52	12.7		11.8
2001–2600	52	12.7		13.5
2601–3600	71	17.4		17.8
3601–5000	71	17.4		16.9
>5000	92	22.5		22.2

Population mean for age and gender based on UN data [37] and income based on Bundeszentrale für politische Bildung [38]. One Person did not identify with male or female.

Note, participants above 70 years old have not been included. The recruitment of participants in the highest income group took a few days longer than other participants, however, we do not expect the delayed data collection to systematically influence results.

## Results

### Acceptance of default nudge designs

**Q1. What design changes improve public acceptance of default nudges for promoting healthy and sustainable food choices?**

Examined changes in the design increased the acceptance of three of the five nudges (see Table 3**)**. First, placing the vegetarian dishes on the first pages of the menu rather than having patrons actively fetch a non-vegetarian menu at the counter (I.e., physical cost) was found to significantly increase the acceptance of the nudge (p(chi²) = 0.00). A similar increase in acceptance was observed for the shift in nudge transparency from a pre-filled, climate-friendly shopping cart to instead offering consumers a choice whether they would prefer a pre-filled grocery cart option (p(chi²) = 0.000), as well as for a shift in the labelling of low energy dishes on the menu from a salience nudge (I.e., bolded text) to an information nudge (I.e., calorie information) (p(chi²) = 0.008). Conversely, no significant difference in acceptance was observed for the examined decrease in the social cost of opting out of the butter nudge at a buffet, nor for the shift to ask consumers whether they would like to donate for regionally produced milk products at checkout rather than actively object to a donation sticker.

**Table 3:** Mean acceptance of default nudge scenarios

Acceptance	N	Mean	Std. Dev.	Full refusal (%)	Indifference (%)	Full acceptance (%)	P-value
N1.1	407	0.246	2.24	20.15	15.72	24.82	
N1.2	408	0.127	2.077	18.14	22.55	19.36	0.1604
N2.1	409	− 0.012	2.2	22.0	17.85	20.29	
N2.2	409	1.509	1.875	8.56	11.25	44.74	0.000
N3.1	409	0.029	2.179	22.74	18.09	18.83	
N3.2	409	0.858	1.876	9.78	24.21	27.87	0.000
N4.1	409	1.438	1.707	5.62	16.38	38.39	
N4.2	409	1.66	1.718	5.62	14.67	48.9	0.008
N5.1	409	0.988	2.104	11.74	13.45	37.9	
N5.2	409	0.968	1.989	11.49	17.6	33.5	0.3853

Test–statistic for the p-values is based on a non-parametric sample test on the equality-of-medians [33]. It tests the null hypothesis that the samples were drawn from populations with the same median

**Fig. 2:**
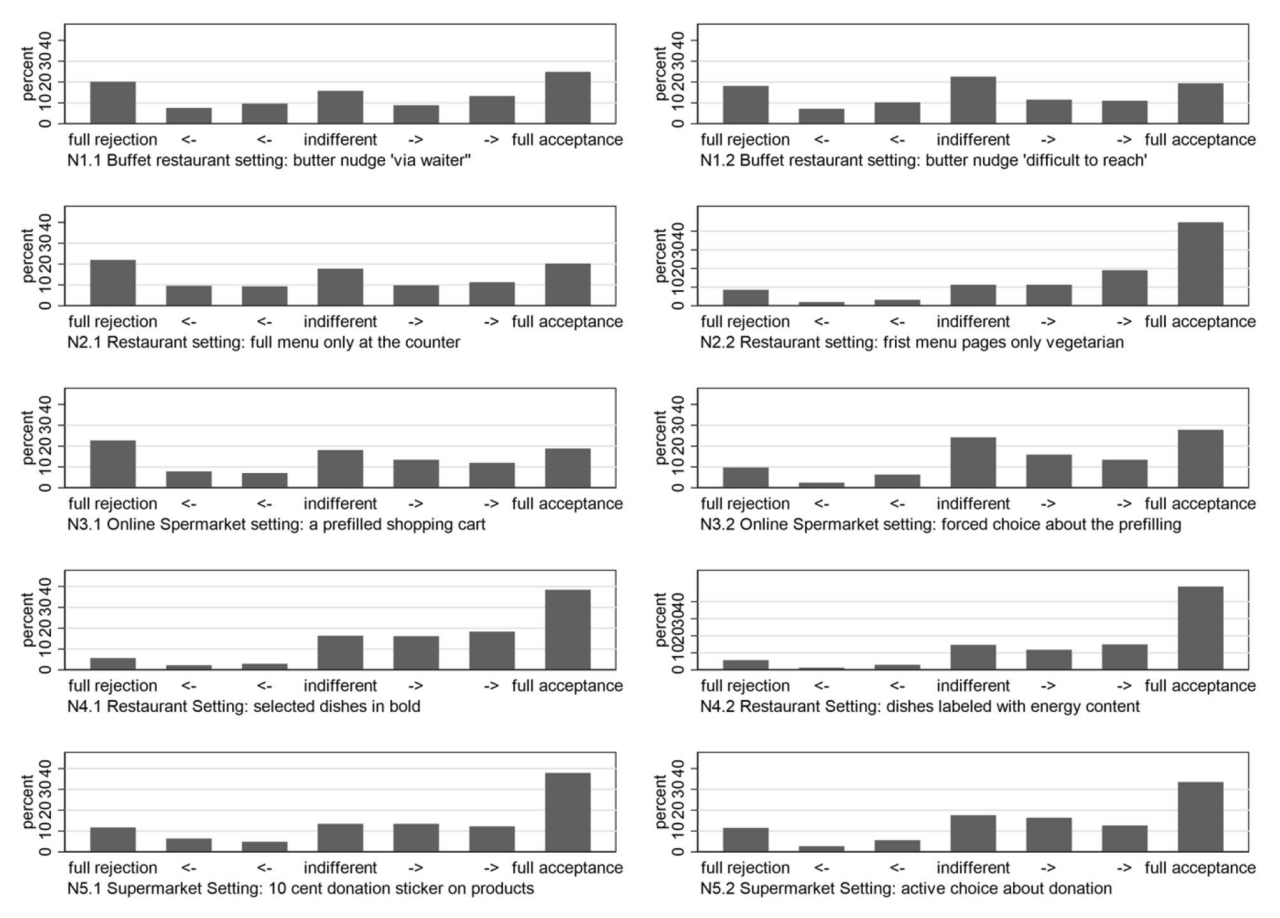
Public acceptance of five nudge designs and their variations. Frequencies in percent (7-point Likert scale) ranging from − 3 “full rejection” to 0 “indifferent” to + 3 “full acceptance”. Description of nudges is summarized in Table 1

Regarding the effect of the design changes on the variation of acceptance, some noteworthy trends can be observed (see Fig. 2). The original iterations of both the second (I.e., default vegetarian menu) and third nudge (I.e., pre-filled online shopping cart) were quite controversial, with 22.0% and 22.8% of participants indicating full refusal and 20.3% and 18.8% indicating full acceptance, respectively. The design change to reduce the physical cost of opting out of the vegetarian nudge is shown to most strongly mitigate nudge controversy, more than halving the share of participants indicating full refusal (-13.4%) and doubling the share of full acceptance (+ 24.5%). The shift in the transparency of the pre-filled online grocery cart nudge was also observed to decrease controversy, but rather by shifting participants towards a higher share of both indifference (+ 6.1%) and full acceptance (+ 9.0%). The first nudge concerning butter accessibility was also highly controversial in its original iteration; however, the proposed design shift to eliminate the social cost of opting out did not significantly mitigate the controversy of the nudge. The fourth and fifth nudges were less controversial to participants than the first three in their original iterations, as each were fully acceptable to a relatively high share of participants in the first place: 38.4% and 37.9%, respectively. For these latter nudges with relatively high acceptance in the beginning, only the shift in menu labelling of side dishes slightly increased full acceptance (+ 10.5%).

### Drivers of default nudge acceptance

**Q2. How do perceived effectiveness, perceived intrusiveness, and engagement in the targeted nudge behavior influence the acceptance of default nudges for promoting healthy and sustainable food choices?**

**Fig. 3:**
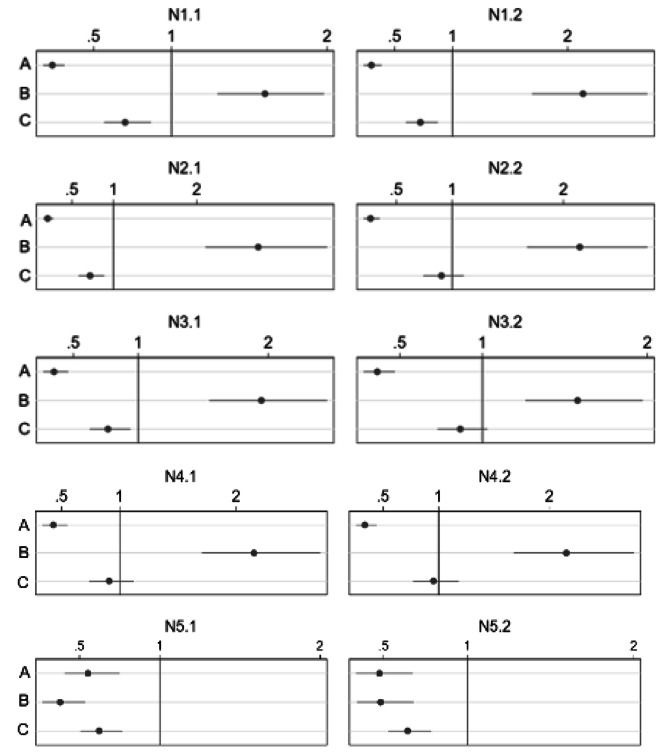
The effect of anticipated drivers on acceptance of studied nudges, expressed as odds ratios (N = 409). **(A)** perceived intrusiveness, **(B)** perceived effectiveness, **(C)** engagement in the target behavior (own behavior). Estimated odds-ratio are displayed with 95% confidence-intervals. All sociodemographic variables are controlled for. For a full regression table, which also includes socio-demographic variables not displayed here, see Supplementary Materials (Table S2)

The perceived intrusiveness of the nudge on individual freedom to choose was found to be the most influential mechanism underpinning acceptance, or lack thereof (see Fig. 3). While the strength of the inverse relationship between perceived intrusiveness and acceptance varied, with the strongest association for the first (OR N1.1 = 0,24; OR N1.2 = 0,30) and second nudges (OR N2.1 = 0,21; OR N2.2 = 0,27), the observed relationship is consistent: the higher the perceived intrusiveness of the nudge on individual freedom, the lower the acceptance. Perceived effectiveness was also found to be a salient driver, with participants indicating higher acceptance of nudges they deemed to be effective at shifting the desired behavior. Engagement in the targeted behavior of the nudge exhibited a negative association with acceptance, though the strength of the association was not comparable to that of either perceived intrusiveness or perceived effectiveness. The fifth nudge is a notable outlier in several respects. First, participants who generally reported higher acceptance of nudges they perceived to be effective reported the opposite for the fifth nudge: the more effective the nudge was perceived to be in increasing donations, the less acceptable it was (OR N5.1 = 0,38; OR N5.2 = 0,48). In addition, those who stated they would donate to support local agriculture found the proposal of a default nudge surrounding this behavior to be less acceptable than those who did not regularly donate (OR N5.1 = 0,62; OR N5.2 = 0,64). Relative to the behavioral and attitudinal mechanisms examined, socio-demographics were observed to carry a small influence on acceptance and were inconsistent in their effect on acceptance across the nudge scenarios.

## Discussion

### On intrusiveness

The results point first and foremost to intrusiveness as a key concept in designing and communicating default nudges that are both effective and acceptable. First, we highlight that the highest increase in acceptance observed across nudge variations pertained to a change in the intrusiveness of the nudge design. Namely, eliminating the physical effort of opting out of a default vegetarian menu transformed a highly contested nudge into a widely accepted one. Second, we highlight the observed preponderance of *perceived* intrusiveness as a key driver of nudge acceptance, or lack thereof. Namely, for all nudges examined, people’s perception of the nudge’s infringement on their individual freedom to choose emerged as the leading factor explaining acceptance, or lack thereof. The importance of perceived intrusiveness is striking, particularly given that existing studies to date examining the effect of shifts in the design and communication of default nudges on acceptance have focused much more squarely on the role of other drivers, such as perceived effectiveness [30, 39] and individual characteristics, such as own behavior [39] and socio-demographics [7]. We therefore posit that there is a salient and under-recognized opportunity for choice architects to calibrate effective and acceptable default nudges by (a) more actively applying design changes to mitigate the costliness of opting out to better preserve individual freedom to choose, as aligned with nudging theory; and (b) actively communicating the preservation of consumer freedom to choose as a central consideration of the nudge design to increase acceptance.

### On effectiveness

Another key concept highlighted in this study is that of effectiveness. The results of this study indicate that concerns of effectiveness and acceptance must be weighed and carefully calibrated for each nudge to discover ‘sweet spots’. For example, removing the social effort of having to ask a waiter for butter in a buffet setting is not found to significantly increase acceptance of the nudge, but it is likely to carry negative consequences for effectiveness, and thus is not a promising design shift for balancing the effectiveness-acceptance trade-off [37]. Conversely, removing the physical effort of deselecting a default vegetarian menu transforms acceptance. While this design change may carry some dilution of effectiveness, it sharply increases the acceptance and thereby the probability of successfully introducing a first nudge in a sustainable direction. In another example, shifting from a salience nudge of low energy side dishes to an information nudge design, specifically calorie labeling, increases acceptance; however, the effect magnitude is just 0.22 on the 7-point acceptance scale, presenting a small difference between two highly accepted nudges. This result is in line with other studies that find such labeling nudges to be among the most acceptable food policies for healthier eating [7, 40]. Thus, effectiveness considerations can be prioritized in this context. Menu labelling policies, highlighted in a recent Cochrane review for their moderate potential to decrease calories consumed in restaurant settings [41], have become increasingly applied, with countries like the U.S. and U.K. introducing mandatory calorie labelling policies for large chain restaurants. Adoption of nudge designs that make healthier choices more salient in food environments, such as increasing the size of healthy options relative to unhealthy choices [42] or shifts in menu positioning of healthy items [43] are relatively less common, though a systematic review of salience nudging studies identified a consistent positive influence for healthier food choices [44]. However, the same systematic review identified a dearth of salience nudges for food choices, pointing to a gap in research and application for adopting potentially effective and acceptable nudges for shifting food choices.

*Perceived* effectiveness is another key aspect of acceptance: consumers need to believe in the intervention’s success in order to prefer it over the status quo [39]. However, the opposite can also be true, as observed in the case of the fifth nudge concerning donations. If the effectiveness of a nudge hinges on a strong form of implied endorsement, which is often described as a psychological mechanism of defaults [26, 40], then people grow particularly wary of effective interventions. A similar result was observed in a cross-country survey in the acceptance of nudges, which noted low acceptance of nudges related to donations, which the authors posit relates to loss aversion: in general, people do not favor default rules that they perceive would take people’s money without their explicit consent [45].

Looking into absolute acceptance values, we highlight the heterogeneity in the responses. We observe with several nudges that a majority identifies with either full rejection or full acceptance. This trend points to the challenges for restaurants, caterers and policy makers to implement effective nudging policies as part of their overall business model or agenda that will be strongly opposed by a substantial share. That said, this study points to one particularly exciting nudge in the context of balancing effectiveness and acceptance. Namely, the fifth nudge, drawn from a real-world policy in Sweden that raised substantial donations for local dairy farmers, demonstrates that a default that clearly does not impose physical effort, substantial time, or money to opt-out of is clearly accepted by most consumers. This real-world example is relevant because policy debates on the transformation of the agricultural and food systems often discuss how to generate money to provide the agricultural sector options to restructure production units. In Germany, the “Borchert Kommission” has introduced several key policies to create a level-playing field for domestic producers when burdening them with additional costs for the transformation. Such nudging policies are not currently considered but could be a way to collect purpose-specific revenues without burdening poorer consumers with additional household spending.

### On transparency

This study also highlights the issue of nudge transparency, which is found to play a significant role in acceptance of a default nudge to shift climate-friendly grocery shopping. Informing consumers about the default option of a pre-packed grocery cart made a substantial difference to acceptance. Transparency is regularly discussed as a key concept to increase the legitimacy of nudges, as it ensures that consumer autonomy is respected [26] and has been studied as a key driver in nudge acceptance [46]. Although transparency can be perceived as paternalistic by some, it also fits well into a world that demands an increasing number of decisions [47], in which such a nudge can help encourage shoppers inclined towards certain behaviors – such as climate friendly or healthy purchases - to more conveniently and effectively live out those values in their shopping behavior. In general, transparent nudges are often similarly effective to non-transparent ones [26, 42, 48], although some context dependencies are still involved. Thus, transparent nudges are generally preferable to non-transparent ones given the similar effectiveness and the edge on acceptance.

### On own behavior

The fourth and final concept touched upon in this study as a driver of nudge acceptance is that of engagement in the targeted behavior of the nudge. The expected deleterious effect of nudges on consumer welfare, such as the costs imposed upon consumers to opt-out of an option that they would regularly reject, is considered a key barrier of nudge acceptance [49]. Previous studies do indicate an association between own engagement in a targeted health behavior and acceptance of policies aimed at changing it [6], such as for interventions related to reducing the consumption of sugary drinks amongst regular consumers [7, 50]. However, such results are characterized by small effect sizes [7] and inconsistency [51].

The results of this study indicate a weak relationship between engagement in the target behavior of the nudge and nudge acceptance. We therefore challenge this straightforward assumption regarding own-behavior and intervention acceptance and recommend giving a low priority to actual behavior while instead considering behavioral intentions of consumers. Indeed, attitudinal factors such as individual sugar consciousness [7, 28] and health consciousness [52] have been found to be drivers of acceptance of a range of healthy eating interventions. Consumers may accept nudges because they feel that they help them to achieve a better version of themselves.

### Limitations and future directions

A key limitation to acknowledge is the study’s focus on a limited set of design elements. While some critical aspects, such as transparency and the cost of opting out, were explored in this study, other potentially influential elements, like customization, were not examined. Furthermore, we did not compare how similar variations in nudge transparency might manifest across different nudge domains. These limitations present opportunities for future research to systematically investigate the relationship between nudge design and acceptance more comprehensively.

Moreover, while our survey did not touch on particularly sensitive topics, it is important to recognize the potential presence of social desirability bias when relying on self-reported measures to assess both mechanisms and acceptance of interventions related to personal well-being and pro-social behaviors [53, 54]. Although complete avoidance of this bias is challenging, we took deliberate steps to maintain neutrality in the language used to describe nudges and other measures, minimizing any implied endorsement of specific behaviors or responses. Additionally, it’s worth noting that each nudge variation was subject to the same systematic bias, allowing for meaningful comparisons within variations but making it challenging to draw causal conclusions when comparing across different nudges due to numerous altered factors.

## Conclusions

This study was driven by two research aims: (1) to identify which design changes improve public acceptance of default nudges for promoting healthy and sustainable food choices; and (2) to examine how attitudinal and behavioral drivers – perceived effectiveness, perceived intrusiveness, and engagement in the targeted nudge behavior - influence the acceptance of default nudges for promoting healthy and sustainable food choices. With regard to the former, the results indicate that mitigating the costliness of opting out and improving the transparency of the nudge are key opportunities for choice architects to improve public acceptance, and thereby potentially identify ‘sweet spots’ in designing default nudges that are both effective and acceptable. With regard to the latter, perceived intrusiveness was found to play the most prominent role in predicting acceptance, followed by perceived effectiveness. Consequently, the protection of individual freedom of choice and effectiveness of default nudging strategies emerge as key aspects for choice architects to communicate to the public to increase acceptance.

### Electronic supplementary material

Below is the link to the electronic supplementary material.

Supplementary Material 1

## Data Availability

The data and analysis code supporting the conclusions of this article are available via GRO.data: 10.25625/XGYIU2. The regression model is available via GitHub: https://github.com/dlemken/Acceptance_default.
