# Supplementary material for: Public acceptance of default nudges to promote healthy and sustainable food choices
Source: BMC Public Health. 2023 Nov 22;23:2311. doi: 10.1186/s12889-023-17127-z (PMC10664270; doi:10.1186/s12889-023-17127-z)
Supplement: Supplementary file 1 — Supplementary Material 1 [file 12889_2023_17127_MOESM1_ESM.docx]

**Supplementary Materials - Public acceptance of default nudges to promote healthy and sustainable food choices**

**Table S1. Statements used to measure mechanisms (own behavior, perceived intrusiveness, perceived effectiveness) of nudge acceptance.**

| Nudge | Item | Statement | Responses |
| --- | --- | --- | --- |
| 1 | Own behavior | Do you normally take butter at a breakfast buffet? | Binary [Rather yes, Rather no] |
|  | Perceived intrusiveness | The described situation restricts your decision. | Seven-point Likert scale |
|  | Perceived effectiveness | The described situation reduces overall butter consumption. | Seven-point Likert scale |
| 2 | Own behavior | What do you usually choose in a restaurant? | Binary [A vegetarian meal, A non-vegetarian meal] |
|  | Perceived intrusiveness | The described situation restricts your decision. | Seven-point Likert scale |
|  | Perceived effectiveness | The described situation reduces overall meat consumption. | Seven-point Likert scale |
| 3 | Own behavior | Do you normally buy dairy or meat products? | Three responses [Rather none, Rather little, Rather a lot] |
|  | Perceived intrusiveness | The described situation restricts your decision. | Seven-point Likert scale |
|  | Perceived effectiveness | The described situation reduces overall consumption of dairy or meat products. | Seven-point Likert scale |
| 4 | Own behavior | Which do you normally order in a restaurant? | Binary [Rather side dishes with low calorie content (salad, vegetables); Rather side dishes with high calories content (fries, mayonnaise)] |
|  | Perceived intrusiveness | The described situation restricts your decision. | Seven-point Likert scale |
|  | Perceived effectiveness | The described situation reduces overall consumption of side dishes with high caloric content. | Seven-point Likert scale |
| 5 | Own behavior | Do you usually donate money to domestic agriculture? | Binary [Rather yes, Rather no] |
|  | Perceived intrusiveness | The described situation restricts your decision. | Seven-point Likert scale |
|  | Perceived effectiveness | The described situation reduces the consumption of regional products in total | Seven-point Likert scale |

**Table S2. Ordinal regression models on the acceptance of a default nudge scenario.** 1.row: odds ratio; 2.row p-values

|  | N1.1 | N1.2 | N2.1 | N2.2 | N3.1 | N3.2 | N4.1 | N4.2 | N5.1 | N5.2 |
| --- | --- | --- | --- | --- | --- | --- | --- | --- | --- | --- |
|  |  |  |  |  |  |  |  |  |  |  |
| perceived intrusiveness | 0.235^***^ | 0.298^***^ | 0.209^***^ | 0.269^***^ | 0.352^***^ | 0.364^***^ | 0.432^***^ | 0.337^***^ | 0.553^***^ | 0.475^***^ |
|  | (0.000) | (0.000) | (0.000) | (0.000) | (0.000) | (0.000) | (0.000) | (0.000) | (0.000) | (0.000) |
| perceived effectiveness | 1.602^***^ | 2.132^***^ | 2.741^***^ | 2.146^***^ | 1.947^***^ | 1.577^***^ | 2.152^***^ | 2.151^***^ | 0.380^***^ | 0.481^***^ |
|  | (0.000) | (0.000) | (0.000) | (0.000) | (0.000) | (0.000) | (0.000) | (0.000) | (0.000) | (0.000) |
| engagement in target behavior | 0.703^**^ | 0.723^***^ | 0.719^**^ | 0.905 | 0.767^*^ | 0.867 | 0.910 | 0.955 | 0.623^***^ | 0.644^***^ |
|  | (0.001) | (0.001) | (0.002) | (0.327) | (0.010) | (0.106) | (0.375) | (0.673) | (0.000) | (0.000) |
| gender | 0.930 | 1.048 | 0.972 | 0.770^**^ | 0.901 | 1.075 | 0.883 | 1.134 | 0.836 | 0.842 |
|  | (0.440) | (0.626) | (0.770) | (0.008) | (0.264) | (0.428) | (0.200) | (0.215) | (0.061) | (0.064) |
| age | 0.908 | 0.951 | 0.889 | 0.842 | 0.802^*^ | 0.838 | 1.004 | 1.029 | 0.693^***^ | 0.817^*^ |
|  | (0.289) | (0.581) | (0.232) | (0.062) | (0.016) | (0.060) | (0.970) | (0.788) | (0.000) | (0.029) |
| East Germany | 0.966 | 0.952 | 1.035 | 0.988 | 0.978 | 0.899 | 1.184 | 1.053 | 1.038 | 1.036 |
|  | (0.715) | (0.569) | (0.720) | (0.897) | (0.774) | (0.264) | (0.059) | (0.590) | (0.688) | (0.673) |
| education | 0.904 | 0.943 | 0.821 | 1.151 | 0.855 | 0.910 | 0.826 | 0.796^*^ | 0.986 | 0.880 |
|  | (0.322) | (0.566) | (0.063) | (0.177) | (0.103) | (0.324) | (0.081) | (0.040) | (0.886) | (0.163) |
| income | 1.075 | 1.154 | 1.180 | 1.076 | 1.178 | 1.181 | 1.306^*^ | 1.239^*^ | 1.025 | 1.194^*^ |
|  | (0.442) | (0.112) | (0.092) | (0.491) | (0.072) | (0.061) | (0.011) | (0.031) | (0.784) | (0.044) |
| Observations | 399 | 400 | 401 | 401 | 401 | 401 | 401 | 401 | 401 | 401 |

^*^ *p* < 0,05, ^**^ *p* < 0,01, ^***^ *p* < 0,001
